# Impact of Smoking and Vaping in Films on Smoking and Vaping Uptake in Adolescents: Systematic Review and Meta-Analysis

**DOI:** 10.1177/10901981221086944

**Published:** 2022-05-03

**Authors:** Zeinab M. Hassanein, Alexander B. Barker, Rachael L. Murray, John Britton, Sanjay Agrawal, Jo Leonardi-Bee

**Affiliations:** 1 University of Nottingham, Nottingham, UK; 2 Assiut University, Egypt; 3 Nottingham Trent University, Nottingham, UK; 4 SPECTRUM Consortium, UK; 5 Glenfield Hospital, Leicester, UK

**Keywords:** smoking initiation, films, movies, vaping, e-cigarettes, systematic review

## Abstract

Prevention of smoking uptake in young people is an essential public health target. We have previously reported a systematic review and meta-analysis of the effect of exposure to smoking imagery in films on the risk of smoking uptake in young people. This study updates that review, and includes studies of the effects of exposure to media vaping imagery on vaping uptake. Four electronic databases (MEDLINE, EMBASE, PsycINFO, and IBSS) were searched to August 2020 for studies reporting the association between exposure to smoking/vaping in films and smoking/vaping uptake in adolescents. Two authors independently screened papers, extracted data, and assessed quality. This review included 26 studies. Exposure to high levels of smoking imagery in films was associated with an increased likelihood of having ever smoked in nine cross-sectional studies and of smoking uptake in 11 longitudinal studies. Vaping imagery in films was associated with increased likelihood of ever vaping in two cross-sectional studies and vaping uptake in five longitudinal studies. This review concluded that exposure to smoking imagery in films increases the risk of smoking among young people. It is likely that a similar association exists between exposure to vaping imagery and vaping uptake. Therefore, this review recommends introduction of new policies to minimize the impact of this in films which contain smoking or vaping and are likely to be viewed by children and adolescents.

Tobacco smoking is a major global cause of preventable diseases and deaths. Smoking currently causes an estimated seven million deaths annually and this number has been expected to double by 2030 (Mathers & Loncar, 2006; World Health Organization, 2017). In 2018, in England, smoking caused approximately 78,000 deaths and half a million hospital admissions (NHS, 2019), imposing a financial burden of £2.5 billion on the National Health Service and a substantially greater financial and amenity loss on society (Action on Smoking and Health, 2018; Goodchild et al., 2018). Since the majority of adults who smoke begin smoking during teenage years (Barrington-Trimis et al., 2020; Public Health England, 2017), preventing smoking experimentation and uptake in young people is a public health priority. E-cigarette use is now common in many countries. In 2019, the e-cigarette use was 27.5% among high school students and 10.5% among middle school students in the United States which lead the U.S. Surgeon General to declare youth e-cigarette use an epidemic (Cullen et al., 2019). In the United Kingdom, 12% of teenagers have tried e-cigarettes (Bauld et al., 2016).

Exposure to tobacco imagery in the films, whether as content or commercial advertising, increases tobacco use by adolescents (Davis et al., 2008; U.S. Department of Health and Human Services, 2012, 2014; World Health Organization, 2016). While paid tobacco advertising and product placement have been prohibited in many countries (Arora et al., 2020; Barker, Whittamore, et al., 2019; Kulkarni et al., 2020; Tynan et al., 2019), tobacco content, branding, and brand alibis, whether paid for or otherwise, still occur in films and other audio-visual media (Barker et al., 2018; Barker, Breton, et al., 2019; Barker, Smith, et al., 2019; Leonardi-Bee et al., 2016; Payne et al., 2016).

In our previously published meta-analysis of eight longitudinal studies published by May 2015, children exposed to high levels of such imagery were found to be nearly 50% more likely to become smokers (Leonardi-Bee et al., 2016) than those unexposed, or exposed to the lowest levels of content. Since carrying out our earlier review (Leonardi-Bee et al., 2016), the evidence base has grown, and the literature is also now beginning to include studies of the association between exposure to vaping imagery and vaping among young people (Camenga et al., 2018; Loukas et al., 2019; Mantey et al., 2016; Pu & Zhang, 2017). Moreover, the social context is changing and the way people consume media has radically changed in the past few years, therefore more recent studies may demonstrate different associations. We have therefore updated our systematic review and meta-analysis of cross-sectional and longitudinal studies of the association between exposure to smoking imagery in films and smoking uptake among young people (Leonardi-Bee et al., 2016), and extended the review to include studies of the effects of vaping imagery. This review is an update of the effect of movie exposure, but that this effect is now even more important than it was, given how much smoking is now present in on-demand video and other media (Barker et al., 2020; Barker, Smith, et al., 2019; Barker, Whittamore, et al., 2019).

## Methods

Our protocol was first registered with the National Institute for Health Research International prospective register of systematic reviews (PROSPERO) under the registration number CRD42014009177 in March 2014. We updated the protocol in 2020 to reflect the broadening of the focus of the review to include exposure to vaping in movies. We adhered to the MOOSE (Stroup et al., 2000) and PRISMA (Moher et al., 2009) guidelines throughout the review.

### Criteria for Considering Studies

We included cross-sectional and longitudinal cohort studies that reported the association between exposure to cigarette smoking or vaping in films and other media forms and smoking or vaping uptake in adolescence (10–19 years; Sawyer et al., 2018). Longitudinal associations were only eligible for inclusion in adolescents who were never smokers/vapers at baseline. We excluded studies in which the average age of the population was older than 19 years; studies which solely focused on exposure to smoking in television programs, series, sitcoms, and trailers; and studies in which smoking susceptibility was the only outcome.

### Search Strategy

Since our previous study (Leonardi-Bee et al., 2016) identified papers published up to May 2015, we performed a comprehensive updated search of four electronic databases from 2015 to August 2020 (MEDLINE, EMBASE, PsycINFO, and International Bibliography of the Social Sciences, IBSS) using controlled vocabulary and text words for smoking initiation, smoking in films, vaping, e-cigarettes and recognized search terms for limiting the searches to specific study designs (Supplemental Table S1; Scottish Intercollegiate Guidelines Network, 2014). No language restrictions were applied for the search results; however, the search strategy was conducted in English. We also searched reference lists of included studies and published reviews to identify further studies.

### Screening and Data Extraction

Papers were screened independently by two authors (ZH and JLB/AB/RM) using a two-stage approach based on (1) titles and abstracts and (2) full text. Any disagreements were resolved through discussion and consensus between authors. No restrictions were placed on language, and translations were sought where necessary.

Data extraction was carried out independently by two authors (ZH and JLB/AB/RM) using a previously piloted data extraction form, which collected information relating to study design, data collection period, definitions of exposure (cigarette smoking in films and vaping in TV/films) and outcome (smoking or vaping uptake), country, setting, inclusion and exclusion criteria for participants, number of participants recruited and evaluated, demographics of study population (age and socio-economic status), quantitative results, and the limitations of the study.

The Newcastle-Ottawa Quality Assessment Scale (Wells et al., 2015) was used to assess the methodological quality of the included studies (maximum score for longitudinal/cohort and cross-sectional studies was nine and seven, respectively), where assessments were made independently by two authors (ZH and JLB/AB/RM), with discrepancies resolved through discussion. A score of six or more was deemed to be high quality (Stang, 2010; Wells et al., 2015).

### Data Synthesis

We estimated pooled relative risks (RR) with 95% confidence intervals (CI) for the effect of exposure to smoking or vaping in films and smoking or vaping uptake, using random effects models. Odds ratios and risk ratios were pooled as relative risks, and we used estimates adjusted for demographic characteristics and/or socio-economic status in preference to unadjusted estimates to minimize the effect of confounding as a source of heterogeneity. We conducted separate pooled analyses for cross-sectional and longitudinal associations but present the pooled estimates for the combination of the two designs for illustrative purposes in the associated Figures. For the meta-analyses, where the exposure to smoking/vaping use in films was reported using categories or quantiles (e.g., tertiles, quartiles, quintiles), we used the most exposed group compared to the least exposed group. Continuous measures of exposure to smoking/vaping in films were used as reported in the publication. Continuous and categorical measures of exposure were pooled together in the meta-analyses. Dose-response association were extracted and reported. Heterogeneity between studies was quantified using I^2^ (Higgins et al., 2003). Where sufficient studies were included in the meta-analysis, subgroup analyses were conducted to explore the reasons for heterogeneity based on study level factors of methodological quality and country. Publication bias was assessed using a funnel plot. The GRADE approach was used to provide an overall assessment of the certainty of the evidence (Guyatt et al., 2008). Each ranking started with a “low-quality” rating because the evidence used was from observational studies. Rankings were upgraded if the magnitude of association was large (RR ≥ 2), there was evidence of a dose response relation, or if all plausible biases would decrease the magnitude of the association; and downgraded if there were serious concerns regarding methodological quality, inconsistency of results, indirectness of evidence, imprecision of result, or reporting bias. *p* values < .05 were deemed statistically significant. Review Manager 5.2 and STATA/MP 13.1 were used to perform analyses.

## Results

**Figure 1.**
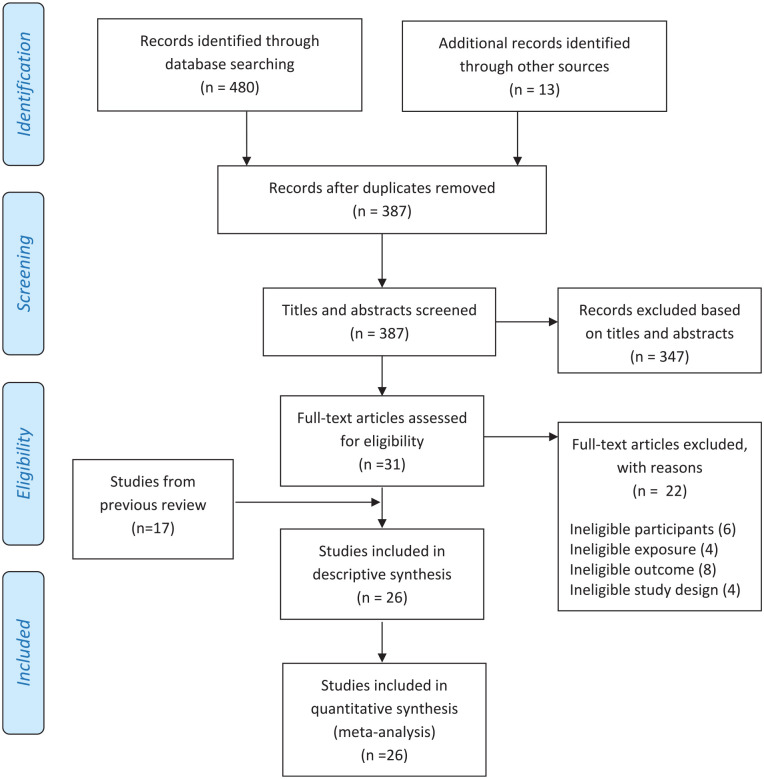
PRISMA flow chart.

From a total of 480 hits that were generated by our searches, 31 were identified as being potentially eligible for inclusion based on their title and abstract. Of these, 22 were excluded because either the participants were ineligible (older than 19 years, six studies), the exposure was ineligible (four studies), the outcome was ineligible (eight studies), or the study design was ineligible (four studies). Adding these nine new studies to the 17 identified in the previous review (Leonardi-Bee et al., 2016), resulted in a total of 26 studies included in this review (Arora et al., 2012; Camenga et al., 2018; Cruz et al., 2019; Dal Cin et al., 2013; Farrelly et al., 2012; Hanewinkel & Sargent, 2007, 2008a; Hansen et al., 2018; Hunt et al., 2009, 2011; Janssen et al., 2018; Loukas et al., 2019; Mejia et al., 2017; Morgenstern et al., 2011, 2013; Nicksic et al., 2017; Pierce et al., 2018; Pu & Zhang, 2017; Sargent et al., 2001, 2005; Sargent et al., 2009; Thrasher et al., 2008, 2009; Titus-Ernstoff et al., 2008; Waylen et al., 2011; Wilkinson et al., 2009; Figure 1). The nine new studies comprised two assessing smoking exposure, six assessing vaping exposure and one assessing both smoking and vaping exposure, in TV/movies.

Among the 26 included studies, 15 were longitudinal in design and 11 cross-sectional. Studies were carried out predominately in one country (the United States, 14 studies; Mexico, two studies; the United Kingdom, three studies; Germany, three studies; India, one study; Argentina, one study), however, two studies were conducted across six European counties (Supplemental Table S2 and S3). All identified studies were published in English. The median sample size was 5,166 (range = 948–21,595) for studies reporting cross-sectional associations, and 2,255 (range = 1,023–9,987) for those reporting longitudinal associations. Twenty studies looked at exposure to smoking in films (nine cross-sectional and 11 longitudinal), two looked at exposure to vaping in films (Cruz et al., 2019; Pu & Zhang, 2017), and five looked at exposure to vaping in other media forms, including TV/movie ads or promotions (Camenga et al., 2018; Hansen et al., 2018; Loukas et al., 2019; Nicksic et al., 2017; Pierce et al., 2018). The median age of the participant populations was 14 years (range = 10–17).

The majority of studies estimated exposure to cigarette smoking in the highest grossing or popular contemporary films using a composite measure based on summing the number of smoking occurrences in single viewings of all the films that participants reported they had seen. The measure of exposure was commonly classified into quantiles; however, eight studies reported exposure as a continuous measure (Cruz et al., 2019; Dal Cin et al., 2013; Farrelly et al., 2012; Hunt et al., 2011; Janssen et al., 2018; Mejia et al., 2017; Titus-Ernstoff et al., 2008; Wilkinson et al., 2009).

In the 11 cross-sectional studies, uptake was defined as either ever tried smoking or ever tried vaping. In the 15 longitudinal studies, uptake was defined as ever use of cigarettes or ever use of vaping reported at follow-up by participants who at baseline had never smoked cigarettes or used vaping, respectively. All of the 26 included studies reported results adjusted for confounders and 14 adjusted for at least one measure of socio-economic status; other common confounders adjusted for included age, sex, school performance, sibling/parental smoking status, parenting style, availability of cigarettes, sensation seeking, type of school, friend’s smoking, and media access (Supplemental Tables S2 and S3).

Twenty-one studies (7/11 cross-sectional, 14/15 longitudinal) were deemed to be of high quality with a Newcastle Ottawa Score ≥6 (Supplemental Tables S2 and S3). None of the included studies met the criteria for ascertainment of exposure, and the majority of studies reporting longitudinal associations did not meet the criterion for ascertainment of outcome, since they relied on self-reporting. No clear evidence of asymmetry (publication bias) was seen in funnel plots for smoking uptake or vaping uptake (Supplemental Figures S1 and S2).

### Cross-Sectional Studies of Ever-Smoking

**Figure 2.**
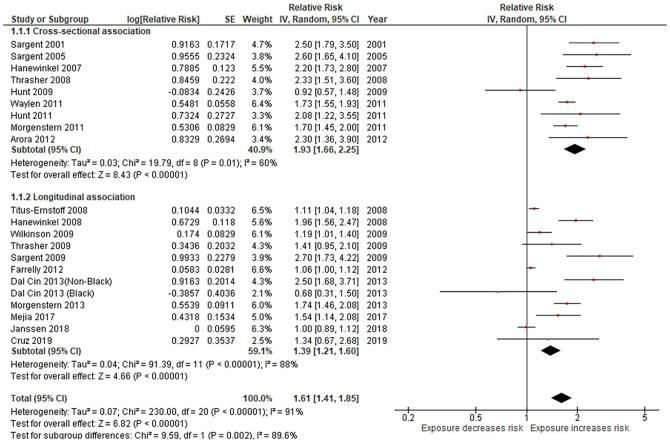
Smoking in movies and smoking uptake among adolescents: cross-sectional and longitudinal studies.

A meta-analysis of effect estimates from the nine cross-sectional studies (Arora et al., 2012; Hanewinkel & Sargent, 2007; Hunt et al., 2009, 2011; Sargent et al., 2001, 2005; Morgenstern et al., 2011; Thrasher et al., 2008; Waylen et al., 2011) found higher exposure to movie smoking was associated with a significantly increased risk of having ever tried smoking (RR = 1.93, 95% CI = 1.66 to 2.25; I^2^ = 60%, Figure 2; moderate certainty evidence; Table 1). All nine studies showed evidence that the strength of this association was exposure-related (Arora et al., 2012; Hanewinkel & Sargent, 2007; Hunt et al., 2009, 2011; Sargent et al., 2001, 2005; Morgenstern et al., 2011; Thrasher et al., 2008; Waylen et al., 2011).

**Table 1.** Assessment of the Certainty of the Evidence.

Outcome	Relative risk (95% CI)	Number of participants (studies)	Quality and justification of ranking (GRADE)	Comments
Smoking uptake				
Longitudinal studies	RR 1.39 (1.21–1.60)	28,554 (11 studies)	⊕⊕⊕ Moderate^a^	Increased by one rank: Evidence of dose response relation in majority of studies
Cross-sectional studies	RR 1.93 (1.66–2.25)	49,521 (nine studies)	⊕⊕⊕ Moderate^a^	Increased by one rank: Evidence of dose response relation in all studies
Vaping uptake				
Longitudinal studies	RR 1.32 (1.13–1.54)	17,562 (five studies)	⊕⊕ Low	No adjustment to rank
Cross-sectional studies	RR 1.36 (1.02–1.81)	28,497 (two studies)	⊕ Very low^b^	Decreased by one rank: Both studies had high risk of bias

a Upgraded due to dose response relation.

b Downgraded due to serious concerns about methodological quality.

### Longitudinal Studies of Smoking Uptake

For the 11 longitudinal studies (Cruz et al., 2019; Dal Cin et al., 2013; Farrelly et al., 2012; Hanewinkel & Sargent, 2008; Janssen et al., 2018; Mejia et al., 2017; Morgenstern et al., 2013; Sargent et al., 2009; Thrasher et al., 2009; Titus-Ernstoff et al., 2008; Wilkinson et al., 2009), higher exposure to movie smoking was associated with an increased risk of smoking uptake among young people, with a relative risk of 1.39 (95% CI = 1.21–1.60, I^2^ = 88%; 11 studies; Figure 2; moderate certainty evidence; Table 1). Seven of the studies showed evidence of an exposure-response relation between increasing exposure to film smoking and increased risk of smoking uptake (Hanewinkel & Sargent, 2007; Mejia et al., 2017; Morgenstern et al., 2013; Sargent et al., 2009; Thrasher et al., 2009; Titus-Ernstoff et al., 2008; Wilkinson et al., 2009).

### Subgroup Analyses

Subgroup analyses based on country found that cross-sectional studies from the United States had a significantly higher pooled estimate than those from elsewhere (the United States: RR = 2.53, 95% CI = 1.93–3.32; Non-U.S.: RR = 1.814, 95% CI = 1.55–2.12; *p* value for subgroup differences = 0.04). However, the reverse was seen for longitudinal studies, with U.S. studies demonstrating a significantly lower pooled estimate than those from elsewhere (the United States: RR = 1.21, 95% CI = 1.07–1.38; Non-U.S.: RR = 1.73, 95% CI = 1.53–1.95; *p* value for subgroup differences < .0001). Subgroup analysis based on methodological quality found no difference between the pooled magnitudes of effect of higher (≥6) and lower (<6) quality studies for cross-sectional studies (*p* value for subgroup differences = .15). Subgroup analysis could not be conducted for longitudinal studies due to all studies having a score ≥6.

### Vaping in Films and Other Forms of Media and Vaping Uptake

**Figure 3.**
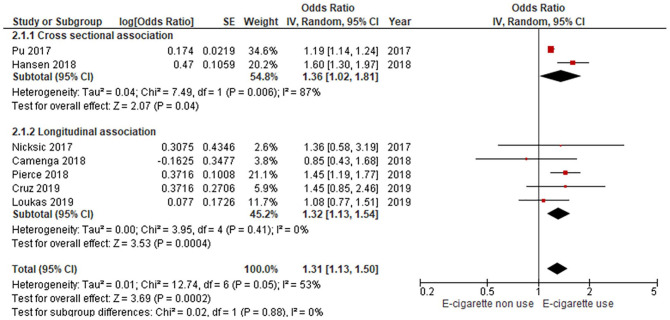
Vaping in TV/movies and vaping uptake among adolescents: cross-sectional and longitudinal studies.

A meta-analysis of effect estimates from the two cross-sectional studies found higher exposure to vaping imagery in television or film was associated with a significantly increased risk of having ever trying vaping (RR = 1.36, 95% CI 1.02–1.81, I^2^ = 87%; Figure 3; very low certainty evidence; Table 1). For the five longitudinal studies, higher exposure to vaping imagery in television or films was associated with a significantly increased risk of vaping uptake among young people by a relative risk of 1.32 (95% CI = 1.13–1.54, I^2^ = 0%; five studies; Figure 3; low-certainty evidence; Table 1). None of the seven studies reported exposure-response associations.

A subgroup analysis of the two cross-sectional vaping studies found that the study conducted in the United States had a lower effect estimate than that carried out in another country (the United States: RR = 1.19, 95% CI = 1.14–1.24; Non-U.S.: RR = 1.60, 95% CI = 1.14–1.24; *p* value for subgroup differences = .006). Subgroup analysis of longitudinal vaping studies based on country was not possible because all identified studies were carried out in U.S. populations.

## Discussion

### Main Finding

As the literature base has grown, this article updates our earlier systematic review and meta-analysis of the effects of exposure to smoking in films and other media on smoking uptake among young people by including nine new studies (Leonardi-Bee et al., 2016) and confirms that even when restricted to studies with longitudinal cohort designs the effect of high relative to low levels exposure is to increase the risk of smoking uptake, by about 40%, with moderate certainty of evidence. This update has identified nine new studies—two assessing smoking exposure, six assessing vaping exposure and one assessing both smoking and vaping exposure, in TV/movies. Since smoking imagery remains prevalent in media accessed by children and young people (Barker et al., 2018; Barker, Breton, et al., 2019; Barker, Smith, et al., 2019; Leonardi-Bee et al., 2016; Payne et al., 2016), this represents a continuing major and completely avoidable influence on smoking uptake and this review is needed to strengthen the evidence base and urge policy makers to consider this evidence. Our study also presents, to our knowledge, the first meta-analysis of studies of the relation between exposure to vaping imagery and vaping uptake and suggests that high levels of exposure may increase uptake of vaping by around 30%.

Inferring causality from observational studies requires circumspection, and particularly so for cross-sectional studies in which temporality between exposure and outcome cannot be guaranteed. Longitudinal studies overcome this concern but remain susceptible to confounding by factors that might increase the likelihood of both exposure to smoking or vaping imagery in films and the uptake of smoking or vaping. While many films containing smoking are given age ratings limiting viewing to older teenagers, it is plausible that the two behaviors are confounded by rebelliousness, though many of the studies we analyzed and adjusted for confounding by “sensation seeking.” There is also evidence that the effect of exposure to film smoking on smoking uptake is greater among children who are otherwise at a relatively low risk of smoking uptake in terms of rebelliousness (Wellman et al., 2016), risk taking and exposure to parental smoking (Hanewinkel & Sargent, 2008; Heatherton & Sargent, 2009; Sargent et al., 2007). This, and the fact that we found consistent evidence of an exposure-response relation adds further weight to the conclusion that the effect of media exposure to smoking is causal. For vaping, the evidence is far less extensive but our finding of an increase risk among exposed children is consistent with that for smoking.

### Strengths and Limitations

The strengths of this systematic review is that it adhered to the PRISMA guidelines, thereby ensuring good conduct and reporting of the systematic review, which included comprehensive searches of a range of electronic databases, without imposing any language restrictions, thereby minimizing the potential of missing eligible studies. This was further reinforced by the absence of evidence of publication bias in the funnel plots. Furthermore, study selection, methodological quality, and data extraction were conducted independently by two authors and we assessed the certainty of evidence using GRADE, which evaluates the confidence that the reported estimates of association and enables stronger recommendations to be drawn for higher quality of evidence.

Due to the nature of the observational study designs, we anticipated that there would be a high level of variation between the estimates of included studies. Thus, we attempted to model this anticipated heterogeneity through using random effects models and minimize the effect of confounding through pooling estimates adjusted for confounders. In addition, we attempted to explore reasons for heterogeneity between studies based on country and methodological quality; however, there was a little variation in the methodological quality of the 26 included studies, with the vast majority having high methodological quality. Although variations in participant level characteristics, such as age, existed, we were unable to explore these effects due to the ecological fallacy. We have updated the search strategy or our previously published systematic review (Leonardi-Bee et al., 2016) to include search terms for vaping for not missing the studies assessing the association with vaping. However, this review has some limitations. The search strategy primarily focused in identifying published studies using electronic databases; therefore, there is the possibility that some unpublished studies may have been omitted since a detailed grey literature search were not conducted. In addition, we only searched for studies with the electronic databases using English language search terms; therefore, we may not have identified all non-English studies. A further limitation is that different cut off points for highest exposure were used within the included studies, which is likely to have resulted in increased heterogeneity between the study estimates, and therefore, may have impacted on widening the 95% confidence interval for the pooled estimate.

### Implications

Viewing habits are changing and new forms of visual media, such as video-on-demand, YouTube, and social media (such as Facebook) are becoming more popular, especially with young people. These services are often unregulated or subject to different regulations that UK films or TV. While research is beginning to explore tobacco content on these digital services (Camenga et al., 2018; Loukas et al., 2019; Mathers & Loncar, 2006; Nicksic et al., 2017; World Health Organization, 2017), future research could explore the effect of adolescent exposure to smoking and vaping imagery in new media forms and its effect on susceptibility or the use of cigarettes or e-cigarettes. Future research may focus on the effect on exposure to advertisements of one product (cigarettes or e-cigarettes) on the use of the other.

Our findings thus provide further updated evidence that exposure to smoking in films causes young people to become smokers, and that it is also likely that exposure to vaping imagery increases the risk of vaping uptake. Whether in this context, vaping represents a diversion from smoking among children who would otherwise have become smokers remains unclear, but our findings do at least provide further support for measures to reduce the exposure of all children to this potentially harmful imagery in films and other media such as video-on-demand, YouTube, and social media (World Health Organization, 2008).

In the United Kingdom, for example, age classification ratings are provided by the British Board of Film Classification (BBFC), whose mission includes protecting the public, and especially children, from content which might cause harm (NHS, 2019). In relation to smoking, BBFC guidelines state only that if smoking features to a significant extent in works which appeal to children, this will be indicated in information provided alongside the age classification and that, despite evidence that the effect of smoking is independent of film character type (“good guy or bad guy”), classification decisions only take into account promotion or glamorization of smoking (World Health Organization, 2017). There are no classification guidelines in regards to vaping. This study shows that smoking and vaping imagery has the potential to lead to uptake, and by not including smoking or vaping imagery in its classification guidelines, the BBFC is not delivering on its mission to protect children from this form of harmful imagery. In future, all films containing smoking and vaping imagery should be assigned an adult (+18) rating to protect children from this content.

For other countries where there is a board responsible for age classification ratings for movies or TV ads and promotions, this board should include vaping imagery in its classification guidelines to protect youth from this harmful imagery that is well established to cause vaping/smoking initiation. Countries all over the world should have a well-designed board which is responsible for age classification rating for movies and its work should be broadened to include TV and other forms of media which is the destination of the youth recently such as video-on-demand, YouTube, and social media.

#### Supplemental Material

sj-docx-1-heb-10.1177_10901981221086944 – Supplemental material for Impact of Smoking and Vaping in Films on Smoking and Vaping Uptake in Adolescents: Systematic Review and Meta-Analysis Supplemental material, sj-docx-1-heb-10.1177_10901981221086944 for Impact of Smoking and Vaping in Films on Smoking and Vaping Uptake in Adolescents: Systematic Review and Meta-Analysis by Zeinab M. Hassanein, Alexander B. Barker, Rachael L. Murray, John Britton, Sanjay Agrawal and Jo Leonardi-Bee in Health Education & Behavior
