# Supplementary material for: Impact of Smoking and Vaping in Films on Smoking and Vaping Uptake in Adolescents: Systematic Review and Meta-Analysis
Source: Health Educ Behav. 2022 May 3;49(6):1004–13. doi: 10.1177/10901981221086944 (PMC9578073; doi:10.1177/10901981221086944)
Supplement: sj-docx-1-heb-10.1177_10901981221086944 – Supplemental material for Impact of Smoking and Vaping in Films on Smoking and Vaping Uptake in Adolescents: Systematic Review and Meta-Analysis [file sj-docx-1-heb-10.1177_10901981221086944.docx]

**Supplementary material**

**Figure S1: Funnel plots for exposure to smoking in movies and smoking uptake**


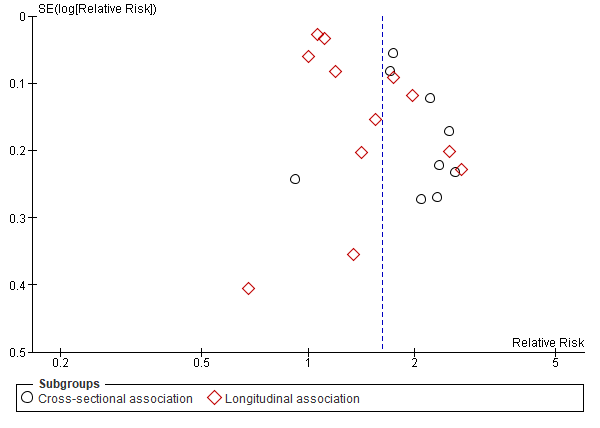


**Figure S2: Funnel plot for exposure to vaping use/imagery in movies and vaping uptake**


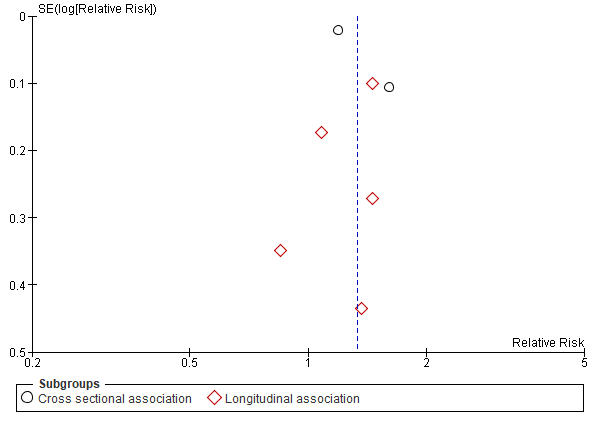


**Table S1: Search strategy terms**

| 1. smok*.mp. or smoking.mp. or exp Smoking/ or tobacco.mp. or exp Tobacco Products/ or exp "Tobacco Use"/ or tobacco.mp. or exp Tobacco/ or nicotine.mp. or nicotine.mp. or exp Nicotine/ or cigarette.mp. or cigar.mp or Electronic Nicotine Delivery Systems/ or Vaping/ or e-cig*.tw,kf or ecig*.tw,kf or electronic cigarette*.tw,kf or (vape* or vaping).tw,kf |
| --- |
| 1. film*.mp. or film.mp. or exp Motion Pictures as Topic/ or movie*.mp. or "motion picture*".mp. or cinema*.mp. |
| 1. longitudinal.mp. or exp Longitudinal Studies/ or longitudinal.mp. or "cohort stud*".mp. or cohort study.mp. or exp Cohort Studies/ or "cohort analysis".mp. or "follow up stud*".mp. or exp Follow-Up Studies/ or retrospective.mp. or exp Retrospective Studies/ or "cross sectional stud*".mp. or exp Cross-Sectional Studies/ |
| 1. 1 and 2 and 3 |

**Table S2 Characteristics of included studies: cross-sectional associations**

| Study (reference) | Country | Sample size, age | Sample of movies, quantiles used in analysis* (dates) | Outcome measures | Outcome data collection period | Metho-dological quality  score | Confounders adjusted for in multivariable analyses |
| --- | --- | --- | --- | --- | --- | --- | --- |
| Cross-sectional associations | | | | | | | |
| Arora 2012 | India | 3,956, 12-16 years | 59 Bollywood top grossing movies, quantiles: 0-86, 87-144, 145-288, >228 (2006-2008) | Ever tried cigarette | 2009 | 5 | Age, gender, school, class, receptive to tobacco advertisements, family smoking, friends smoking, social influences, academic performance, sensation seeking, authoritative parenting |
| Hanewinkel 2007 | Germany | 5,586, 10-17 years | 50 out of 398 top box office movies, quantiles: ≤167, 168-423, 424-801, ≥802 (1994-2004) | Ever tried cigarette | 2005 | 6 | Age, sex, parental smoking, sibling smoking, friend smoking, school performance, school, sensation seeking/rebelliousness, television, DVD and video consumption during the week and at the weekend, receptivity to tobacco marketing, parenting style |
| Hansen 2018 | Germany | 6,902, 11-15 years | 3 top popular grossing vaping TV ads (February–April 2016), Never exposed to vaping ads versus exposed to at least one ad. | Ever tried vaping | 2016-2017 | 5 | Age, sex, migration background, school type, consumer susceptibility to interpersonal influence, socioeconomic status, sensation seeking, and vaping use of at least one friend |
| Hunt 2009 | Scotland | 948,  11-19 years | 50 out of 601 popular contemporary and box office movies, quantiles: 1-139, 140-201, 202-286, >287 (1998-1999) | Ever tried cigarette | 2002-2004 | 6 | Gender, parent social class, parent smoking, risk behaviour, education, peer smoking |
| Hunt 2011 | Scotland | 1,999, 15-16 years | 50 out of 368 top box office movies, quantiles: analysed as continuous measure (2001-2006), Beach method, 95 percentile is high exposure to smoking in films | Ever tried cigarette | 1999 | 6 | Sex, Television/film parenting scale, internet usage supervision, film viewing patterns, housing tenure, parental education, family connectedness, parental monitoring, number of national exams being sat, school leaving plans, peer smoking, views films with friends |
| Morgenstern 2011 | 6 European countries | 16,551, 10-19 years | 50 out of 250 top box office movies, quantile: definitions not reported (2004-2009) | Ever tried cigarette | 2009-2010 | 6 | Age, sex, family affluence, school performance, television screen time, number of movies seen, sensation seeking, rebelliousness, parental smoking, sibling smoking, friend smoking, country, school, class |
| Pu 2017 | United States | 21,595, 12-16 years | Self-reported exposure to ads or promotions for vaping during using TV/movies (2014), Those who reported ‘sometimes’, ‘most of the time’, or ‘always’ were classified as having been exposed to e-cigarette advertising. | Ever tried vaping | 2014 | 5 | Age, gender, race, and cigarette smoking status |
| Sargent 2001 | United States | 4,919, 9-15 years | 50 out of 603 box office movies, quantiles: 0-50, 51-100, 101-150, >150 (1988-1999) | Ever tried cigarette | 1999 | 6 | Age, sex, parents’ education, school, friend smoking, sibling smoking, parent smoking, receptivity to tobacco promotions, school performance, propensity to sensation seeking, rebelliousness, authoritative parenting, perception of parent disapproval of smoking |
| Sargent 2005 | United States | 6,522, 10-14 years | 50 out of 532 top box office movies, quantiles: <19, 19-45, 46-87, ≥88 (1998-2002) | Ever tried cigarette | 2003 | 5 | Age, sex, race, parents’ education, peer smoking, parent smoking, sibling smoking, school performance, sensation seeking, rebelliousness, self-esteem, parenting style |
| Thrasher 2008 | Mexico | 3,874, 11-16 years | 42 out of 165 top grossing movies with at least one minute of smoking content, quantiles: ≤22.83, 22.84-47.92, 47.93-74.13, ≥74.13 minutes of tobacco content (2000-2005) | Ever tried cigarette | 2006 | 6 | Age, sex, sensation seeking, self-esteem, parental smoking, sibling smoking, best friend smoking, television in bedroom, school, bogus films watched |
| Waylen 2011 | UK | 5,166, 7-13 years | 50 out of 306 top box office movies, quantiles: ≤38, 39-68, 69-108, ≥109 (2001-2005) | Ever tried cigarette | 2006-2007 | 6 | Age, sex, social class, financial difficulties, housing, maternal age, maternal education, marital status, maternal smoking, parity, partner smoking, breast feeding, parental monitoring |

**Table S3 Characteristics of included studies: Longitudinal associations**

| Study (reference) | Country | Sample size, age | Sample of movies, quantiles used in analysis* (dates) | Outcome measures | Outcome data collection period | Metho-dological quality  score | Confounders adjusted for in multivariable analyses |
| --- | --- | --- | --- | --- | --- | --- | --- |
| Longitudinal associations | | | | | | | |
| Camenga 2018 | United States | 1,742, 12-16 years | Self-reported exposure to ads or promotions for vaping in television/radio, (seen versus non-seen) | Vaping uptake | 2013- 2014 | 5 | Age, sex, race, cigarette smoking status |
| Cruz 2019 | United States | 1,553, 15-19 years | Self-reported exposure to vaping promotion on watching TV or going to the movies (2014-2016), high exposure included who reported “most of the time” and “always” exposure of vaping in TV/movies | Vaping uptake | 2014-2016 | 6 | Gender, ethnicity, parents’ education, tobacco product use, community |
| Dal Cin 2013 | United States | 2,341, 13-19 years | 50 out of 383 Black orientated and mainstream movies, quantiles: analysed as continuous measure (dates not reported) | Cigarette smoking uptake | 2007- 2009 | 7 | Age, sex, socioeconomic status, conduct disorder symptoms, sensation seeking, peer and sibling smoking, parental responsiveness and monitoring, hours of television per day, presence of television in bedroom |
| Farrelly 2012 | United States | 1,511, 13-16 years | 30 top grossing movies selected based on having smoking occurrences, quantiles: analysed as continuous measure (2004-2007), High exposure is the highest quartile of exposure to smoking in the  movies | Cigarette smoking uptake | 2005-2008 | 6 | Age, sex, race, residence, school, academic achievement, adults at home after school, employment, income, church attendance, friend smoke tobacco, friend smoke marijuana, exposure to second-hand smoke, presence of smoking ban in household, exposure to tobacco use prevention lessons in school, sensation seeking, receptivity to tobacco marketing, parental monitoring of and rules about watching R-rated movies |
| Hanewinkel 2008 | Germany | 2,711, 10-16 years | 50 out of 383 box office movies, quantiles: 0-89, 90-279, 280-580, >581 (1994-2004) | Cigarette smoking uptake | 2005 | 7 | Age, sex, school, parent smoking, sibling smoking, friend smoke, school performance, favourite tobacco advertisement, sensational seeking/ rebelliousness, parenting style |
| Janssen 2018 | United States | 1,023, 10-15 years | 50 out of 500 top-rated movies, quantiles: analysed as continuous exposure (2009-2013) | Cigarette smoking uptake | 2009-2013 | 7 | Age, gender, race, ethnicity, cigarette availability, peer smoking, parental smoking |
| Loukas 2019 | United States | 2,288, 12-17 years | Self-reported exposure to vaping marketing in TV (2017-2019) , (seen versus non-seen) | Vaping uptake | 2017-2019 | 7 | Sex, race and/or ethnicity, grade level, past-30-day or current other tobacco use, sensation seeking, and peer ENDS use |
| Mejia 2017 | Argentina | 1,700, 11-16 years | 50 out of 422 highest-earning Hollywood and Argentine films ), quantiles: analysed as continuous exposure (2009-2013) | Cigarette smoking uptake | 2014-2015 | 7 | Age, sex, parent education, type of school, sensation seeking, parenting style, friends smoking, household smoking, and media access. |
| Morgenstern 2013 | 6 European countries | 9,987, 13-15 years | 50 box office movies, quantiles: definitions not reported (dates not reported) | Cigarette smoking uptake | 2011 | 7 | Age, sex, family affluence, school performance, television screen time, sensation seeking, peer smoking, sibling smoking, parental smoking, country, school, class |
| Nicksic 2017 | United States | 2,488, 12-17 years | Self-reported exposure to ads or promotions for vaping in TV, , (seen versus non-seen) (2014–2015) | Vaping uptake | 2014–2015 | 7 | Sex, race/ethnicity, grade level, and current other tobacco use |
| Pierce 2018 | United States | 9,491, 12-17 years | 20 out of 959 near-census collection of ads (including television ads) for vaping quantiles: analysed as continuous exposure (2013-2015) | Vaping uptake | 2013 | 7 | Sex , race/ethnicity, age, and exposure to smoking in household and social settings |
| Sargent 2009 | United States | 2,603, 10-14 years | 50 out of 601 popular contemporary movies, quantiles: definitions not reported (dates not reported) | Cigarette smoking uptake | 1999 | 7 | Age, sex, school, parents’ education, parental smoking, sibling smoking, friend smoking, school performance, sensation seeking, rebelliousness, self-esteem, maternal demandingness, maternal responsiveness, parental disapproval of smoking |
| Thrasher 2009 | Mexico | 1,741, 11-14 years | 42 out of 165 top grossing movies with at least one minute of smoking content, quantiles: <17.9, 17.9-39.5, 39.5-64.3, >64.3 minutes of tobacco content (2000-2005) | Cigarette smoking uptake | 2006 | 7 | Age, sex, parent smoking, sibling smoking, best friend smoking, parental punishment for smoking, parental authority, own something with tobacco branding, school, self-esteem, sensation seeking, bogus films watched |
| Titus-Ernstoff 2008 | United States | 2,255, 9-12 yrs | 50 out of 550 popular contemporary movies, quantiles: analysed as continuous measure (1997-2003) | Cigarette smoking uptake | 2002-2003 | 7 | Age, sex, race, school performance, self-esteem, self-regulation, rebelliousness, sensation seeking, parental education, parental smoking, maternal monitoring, maternal responsiveness, friend smoking |
| Wilkinson 2009 | United States | 1,129, 11-13 yrs | 50 out of 250 top box office movies, quantiles: analysed as continuous exposure (1999-2004) | Cigarette smoking uptake | 2001 | 7 | Age, sex, friend smoking, risk taking tendencies, detentions at school |

* Exposure variable was measured as the number of smoking occurrences in movies, unless otherwise specified

**PRISMA checklist**

| **Section/topic** | | **#** | | **Checklist item** | **Reported on page #** |
| --- | --- | --- | --- | --- | --- |
| **TITLE** | | | | |  |
| Title | | 1 | | Identify the report as a systematic review, meta-analysis, or both. | 1 |
| **ABSTRACT** | | | | |  |
| Structured summary | | 2 | | Provide a structured summary including, as applicable: background; objectives; data sources; study eligibility criteria, participants, and interventions; study appraisal and synthesis methods; results; limitations; conclusions and implications of key findings; systematic review registration number. | 2 |
| **INTRODUCTION** | | | | |  |
| Rationale | | 3 | | Describe the rationale for the review in the context of what is already known. | 3 |
| Objectives | | 4 | | Provide an explicit statement of questions being addressed with reference to participants, interventions, comparisons, outcomes, and study design (PICOS). | 3 |
| **METHODS** | | | | |  |
| Protocol and registration | | 5 | | Indicate if a review protocol exists, if and where it can be accessed (e.g., Web address), and, if available, provide registration information including registration number. | 4 |
| Eligibility criteria | | 6 | | Specify study characteristics (e.g., PICOS, length of follow-up) and report characteristics (e.g., years considered, language, publication status) used as criteria for eligibility, giving rationale. | 4 |
| Information sources | | 7 | | Describe all information sources (e.g., databases with dates of coverage, contact with study authors to identify additional studies) in the search and date last searched. | 4 |
| Search | | 8 | | Present full electronic search strategy for at least one database, including any limits used, such that it could be repeated. | Table S1 |
| Study selection | | 9 | | State the process for selecting studies (i.e., screening, eligibility, included in systematic review, and, if applicable, included in the meta-analysis). | 5 |
| Data collection process | | 10 | | Describe method of data extraction from reports (e.g., piloted forms, independently, in duplicate) and any processes for obtaining and confirming data from investigators. | 5 |
| Data items | | 11 | | List and define all variables for which data were sought (e.g., PICOS, funding sources) and any assumptions and simplifications made. | 5 |
| Risk of bias in individual studies | | 12 | | Describe methods used for assessing risk of bias of individual studies (including specification of whether this was done at the study or outcome level), and how this information is to be used in any data synthesis. | 5 |
| Summary measures | | 13 | | State the principal summary measures (e.g., risk ratio, difference in means). | 6 |
| Synthesis of results | | 14 | | Describe the methods of handling data and combining results of studies, if done, including measures of consistency (e.g., I^2^) for each meta-analysis. | 6 |
| Risk of bias across studies | 15 | | Specify any assessment of risk of bias that may affect the cumulative evidence (e.g., publication bias, selective reporting within studies). | | 6 |
| Additional analyses | 16 | | Describe methods of additional analyses (e.g., sensitivity or subgroup analyses, meta-regression), if done, indicating which were pre-specified. | | 6 |
| **RESULTS** | | | | |  |
| Study selection | 17 | | Give numbers of studies screened, assessed for eligibility, and included in the review, with reasons for exclusions at each stage, ideally with a flow diagram. | | 7, Fig 1 |
| Study characteristics | 18 | | For each study, present characteristics for which data were extracted (e.g., study size, PICOS, follow-up period) and provide the citations. | | 7 ,Table 1 |
| Risk of bias within studies | 19 | | Present data on risk of bias of each study and, if available, any outcome level assessment (see item 12). | | 8, Figures 2 and 3 |
| Results of individual studies | 20 | | For all outcomes considered (benefits or harms), present, for each study: (a) simple summary data for each intervention group (b) effect estimates and confidence intervals, ideally with a forest plot. | | 8,9, Fig 2, 3 |
| Synthesis of results | 21 | | Present results of each meta-analysis done, including confidence intervals and measures of consistency. | | 7-9,Fig 2,3 |
| Risk of bias across studies | 22 | | Present results of any assessment of risk of bias across studies (see Item 15). | | 8, Figures S1,S2 |
| Additional analysis | 23 | | Give results of additional analyses, if done (e.g., sensitivity or subgroup analyses, meta-regression [see Item 16]). | | 9 |
| **DISCUSSION** | | | | |  |
| Summary of evidence | 24 | | Summarize the main findings including the strength of evidence for each main outcome; consider their relevance to key groups (e.g., healthcare providers, users, and policy makers). | | 10 |
| Limitations | 25 | | Discuss limitations at study and outcome level (e.g., risk of bias), and at review-level (e.g., incomplete retrieval of identified research, reporting bias). | | 11 |
| Conclusions | 26 | | Provide a general interpretation of the results in the context of other evidence, and implications for future research. | | 12 |
| **FUNDING** | | | | |  |
| Funding | 27 | | Describe sources of funding for the systematic review and other support (e.g., supply of data); role of funders for the systematic review. | | 1 |
